# Imported case of avian influenza A(H9N2) virus infection in a patient with miliary tuberculosis, Italy, March 2026

**DOI:** 10.2807/1560-7917.ES.2026.31.15.2600285

**Published:** 2026-04-16

**Authors:** Elena Pariani, Simona Puzelli, Gabriele Del Castillo, Greta Romano, Luca Mezzadri, Cristina Galli, Irene Maria Sciabica, Luigi Vezzosi, Francesca Sabbatini, Cristina Paduraru, Irene Mileto, Marcello Tirani, Anna Teresa Palamara, Paola Stefanelli, Fausto Baldanti, Danilo Cereda, Paolo Bonfanti, Collaborating Centres’ Study Group on Influenza, Collaborating Centres’ Study Group on Influenza, Sandro Binda, Valeria Primache, Emanuela Matteucci, Arlinda Seiti, Michela Marcella Colleoni, Elisa Colella, Alban Rugova, Selma El Ouardi, Giuseppe Lapadula, Viola Cogliandro, Guglielmo Marco Migliorino, Martina Comolatti, Eleonora Maria Beretta, Annalisa Cavallero, Sergio Malandrin, Iacopo Franconi, Dario Lafranceschina, Marzia Facchini, Angela Di Martino, Sara Piacentini, Emanuela Giombini, Giuseppina Di Mario, Concetta Fabiani, Simone Villa, Manuel Maffeo, Simona Scarioni, Marco Campana, Maria Virginia Coscarelli, Emanuele De Ponti, Anna Carole D’Amelio, Angela Ancona, Andrea Pedot, Federica Attanasi, Michela Viscardi, Gherard Batisti Biffignandi, Alessandro Ferrari, Guglielmo Ferrari, Stefano Gaiarsa, Antonio Piralla, Antonino Maria Guglielmo Pitrolo, Francesca Rovida, Raffaele Bruno, Valentina Zuccaro, Andrea Gori, Riccardo Giorgi, Stefania Merli, Alberto Dolci, Alessandra Lombardi, Davide Mileto, Alessandro Mancon, Alberto Rizzo, Valeria Micheli, Chiara Ardemagni, Marianna Denova, Antonina Ilardo

**Affiliations:** 1 Department of biomedical sciences for health, University of Milan, Milan, Italy; 2 National Influenza Centre, Department of Infectious Diseases, Istituto Superiore di Sanità, Rome, Italy; 3 Department of Prevention, General Directorate for Health, Lombardy Region, Milan, Italy; 4 Regional Centre for Infectious Diseases, Lombardy Region (Ceremi), Italy; 5 Microbiology and Virology Department, Fondazione IRCCS Policlinico San Matteo, Pavia, Italy; 6 Infectious Diseases Unit, Fondazione IRCCS San Gerardo dei Tintori, Monza, Italy; 7 School of Medicine and Surgery, University of Milano-Bicocca, Milan, Italy; 8 Microbiology Unit, Fondazione IRCCS San Gerardo dei Tintori, Monza, Italy; 9 Health Protection Agency of Monza-Brianza, Monza, Italy; 10 Department of Clinical, Surgical, Diagnostic and Paediatric Sciences, University of Pavia, Pavia, Italy; 11 The members of the Collaborating Centres’ Study Group on Influenza are listed under Collaborators

**Keywords:** A(H9N2), avian influenza, pandemic preparedness, surveillance, virus, public health response

## Abstract

On 21 March 2026, avian influenza A(H9N2) virus was confirmed in Italy in a patient with miliary tuberculosis. The patient had recently travelled to West Africa. Following the detection of an unsubtypable influenza A virus, rapid molecular confirmation and full genome sequencing were performed. Phylogenetic analysis revealed that the virus belonged to subclade G5.5 and was closely related to African strains. Epidemiological investigations identified no additional cases, suggesting there was no evidence of onward transmission at the time of reporting.

In March 2026, avian influenza A(H9N2) virus was identified in Italy in a patient with weakened immune system. They had recently travelled to West Africa, which raised concerns about the potential importation of zoonotic influenza viruses into Europe, as H9N2 has been endemic in poultry across the region since 2017, with widespread outbreaks and two human cases reported in Senegal (one in 2020) and Ghana (one in 2024) [1,2]. Here we present the results of the virological and epidemiological investigation of this case, including molecular characterisation of the virus and an assessment of the likelihood of onward transmission.

## Case description and virological findings

In mid-March 2026, an adult patient presented to the emergency department of our hospital, major tertiary referral centre in the Lombardy Region, Italy. They had experienced fever and cough since mid-January, accompanied by notable weight loss. They had returned from Senegal on the day of admission, having stayed there for more than 6 months [2]. The patient did not seek medical care or take any medication during their stay in West Africa. They recognised and self-monitored fever. Upon arrival, they were clinically stable, with an oxygen saturation of 97% on room air and a body temperature of 38.1°C. Laboratory findings showed anaemia, hyponatraemia and elevated lactate dehydrogenase (Table 1). A nasal-pharyngeal swab (NPS) tested negative for severe acute respiratory syndrome coronavirus 2 (SARS-CoV-2), influenza A virus (IAV), influenza B virus and respiratory syncytial virus (RSV) (Table 2). A chest X-ray showed consolidation in the right middle and lower lung fields, and a small pleural effusion. A chest computer tomography (CT) scan revealed extensive consolidation of the left upper lobe, diffuse bilateral micronodules and a large right pleural effusion. An abdominal CT scan showed multiple hypodense lesions on the spleen and moderate ascites. As miliary tuberculosis was suspected, the patient was admitted to a single negative-pressure isolation room under airborne isolation precautions. Two days after admission, analysis of a sample from bronchoalveolar lavage (BAL) confirmed the presence of *Mycobacterium tuberculosis*. Anti-tuberculosis therapy comprising rifampicin, isoniazid, ethambutol and pyrazinamide was initiated. Further immunological evaluation revealed considerable cellular immunosuppression (Table 1).

**Table 1:** Clinical and laboratory findings of a patient with *Mycobacterium tuberculosis* and avian influenza A(H9N2) virus co-infection, Italy, March 2026

Parameter	Reference range	Day 0	Day 4	Day 7	Day 12	Day 16	Day 30
Vitals							
Temperature (°C)		38.1	39.4	36.5	38.2	39	39.5
Oxygen saturation (% on RA)		97	94	96	99	99	99
Respiratory rate (breaths/min)		16	29	17	18	18	17
Blood pressure (mmHg)		112/80	116/77	120/80	120/80	110/80	117/77
Haematological parameters							
Haemoglobin (g/L)	142–172	78	81	83	89	93	97
White blood cells (× 10^9^/L)	4–9	5.43	4.72	5.45	4.78	3.19	6.67
Neutrophils (× 10^9^/L)	2–6.2	2.90	3.50	3.58	2.77	2.05	4.87
Lymphocytes (× 10^9^/L)	1.12–3-37	2.07	1.04	1.39	1.37	0.82	0.98
Platelets (× 10^9^/L)	155–340	156	162	197	277	271	300
Inflammation markers							
C-reactive protein (mg/L)	< 5	59.5	33.3	53.8	18.6	28.0	39.6
Procalcitonin (µg/L)	< 0.5	1.27	0.87	NA			
Ferritin (µg/L)	30–400	9,170	NA	NA	3,917	NA	NA
Biochemical parameters							
Creatinine (mg/dL)	0.67–1.17	0.7	0.7	0.6	0.6	0.6	0.6
AST (U/L)	14–35	92	78	64	63	64	64
ALT (U/L)	9–59	36	35	29	27	28	27
LDH (U/L)	135–225	414	339	339	313	354	335
Total bilirubin (mg/dL)	< 1.4	0.7	0.9	0.7	0.6	0.6	0.6
Sodium (mmol/L)	136–145	129	130	131	131	133	131
Potassium (mmol/L)	3.5–5.1	4.0	4.1	4.4	4.7	4.2	4.2
Coagulation							
INR		1.3	NA	1.2	NA		
Immunological parameters							
CD4^+^ T-cells (cells/mm^3^)	500–1,500	150	NA				
CD8^+^ T-cells (cells/mm^3^)	200–800	470					
NK cells (CD16^+^/56^+^) (cells/mm^3^)	90–600	150					
Microbiology							
Testing for hepatitis viruses							
HBV surface antigen (IU/mL)		2,800	NA				
HBV surface antibodies		Negative					
HBV core antibodies		Positive					
HBV e antigen		Negative					
HBV e antibodies		Positive					
HBV DNA (IU/mL)		2.4 × 10^6^					
HCV serology		Negative					
Testing for syphilis							
TP antibodies		Positive	NA				
RPR		Negative					
Other microbiological analyses							
IGRA (QuantiFERON)		Positive	NA				
Blood culture		Negative	NA	Negative	NA		

ALT: alanine transaminase; AST: aspartate aminotransferase; HBV: hepatitis B virus; HCV: hepatitis C virus; IGRA: interferon gamma release assay; INR: international normalised ratio; LDH: lactate dehydrogenase; NA: not analysed; RA: room air; RPR: rapid plasma reagin; TP: *Treponema pallidum*.

**Table 2:** Results of molecular testing for the detection and subtyping of influenza A virus, March 2026

Specimen	Days of sampling post admission	Days of analysis post admission	Testing laboratory	Molecular test	Result
NPS	Day 0	Day 0	HML	Alinity m resp-4-plex assay^a^	IAV: UD
BAL	Day 2	Day 2	HML	Allplex Respiratory Panel 1A^b^	IAV: Cq = 24.6 H1pdm09: UD H3: UD
BAL	Day 2	Day 2	HML	Alinity m resp-4-plex assay^a^	IAV: Cq = 17
BAL	Day 2	Day 5	RRL	CDC real-time RT-PCR protocol^c^	IAV: Cq = 19.62 H1pdm09: UD H3: UD
BAL	Day 2	Day 5	RRL	Viasure Flu Typing II^d^	H1pdm09: UD H3: UD H5N1: UD H7N9: UD
BAL	Day 2	Day 6	RRL	Real-time RT-PCR for influenza A/H5, A/H7, A/H9 detection^e^	H5: UD H7: UD H9: Cq = 19.52
NS	Day 6	Day 6	HML	Alinity m resp-4-plex assay^a^	IAV: UD
TS	Day 6	Day 6	HML	Alinity m resp-4-plex assay^a^	IAV: Cq = 37.39
BAL	Day 2	Day 7	NIC	CDC real-time RT-PCR protocol^c^	IAV: Cq = 17.6 H1pdm09: UD H3: UD
BAL	Day 2	Day 7	NIC	Real-time RT-PCR protocol for influenza A/H5 detection (A/H5a – A/H5b subtyping)^f^	H5a: UD H5b: UD
BAL	Day 2	Day 7	NIC	Real-time RT-PCR for avian influenza viruses of H9 subtype^g^	H9: Cq = 17.2
Pleuric liquid	Day 12	Day 12	HML	Alinity m resp-4-plex assay^a^	IAV: UD
NPS	Day 12	Day 12	HML	Alinity m resp-4-plex assay^a^	IAV: Cq = 33.25
Pleuric liquid	Day 12	Day 12	RRL	CDC real-time RT-PCR protocol^c^	IAV: UD
NPS	Day 12	Day 12	RRL	Real-time RT-PCR for influenza A/H9 detection^e^	H9: Cq = 37.25
BAL	Day 16	Day 16	HML	Alinity m resp-4-plex assay^a^	IAV: Cq = 19.52
BAL	Day 16	Day 17	RRL	Real-time RT-PCR for influenza A/H9 detection^e^	H9: Cq = 21.73

BAL: bronchoalveolar lavage; CDC: Centers for Disease Control and Prevention; Cq: quantification cycle; HML: hospital microbiology laboratory; IAV: influenza A virus; NIC: National Influenza Centre; NPS: nasal-pharyngeal swab; NS: nasal swab; RRL: regional reference laboratory; TS: throat swab; UD: undetermined; US: the United States.

^a^ Multiplex assay for detection of influenza A and B viruses, respiratory syncytial virus and SARS-CoV-2, manufactured by Abbott Molecular Inc., Des Plaines, US.

^b^ Flu/RSV/Flu A subtyping, manufactured by Seegene Inc., Seoul, South Korea.

^c^ US CDC protocol for detection of influenza A (M gene) and seasonal H1/H3 (HA gene) subtyping [19].

^d^ Real-time PCR test for identification and differentiation of H1N1, H3N2, H5N1 and H7N9, manufactured by Certest Biotec, S.L. Zaragoza, Spain.

^e^ Real-time RT-PCR for detection of A(H5N1) clade 1/2/3, A(H7N9), A(H9N2) viruses [20].

^f^ US CDC protocol for detection of influenza A/H5 (A/H5a – A/H5b subtyping) [19].

^g^ Real-time RT-PCR protocol for avian influenza viruses of the H9 subtype [21].

The sample from BAL was tested using two commercial multiplex assays for respiratory virus detection, revealing a positive result for IAV. However, the H1pdm09 and H3 subtyping assays were negative (Table 2). According to the regional pandemic preparedness plan for influenza, all respiratory samples testing positive for IAV but negative for the seasonal subtypes should be sent immediately to a regional reference laboratory (RRL). There are three RRLs in the Lombardy region: the University of Milan, the Fondazione IRCCS Policlinico San Matteo and the ASST Fatebenefratelli-Sacco [3]. Further real-time RT-PCR testing at the University of Milan RRL confirmed the presence of IAV, with no detection of the H1pdm09 or H3 seasonal subtypes or avian A(H5N1) or A(H7N9). Given the suspicion of a zoonotic IAV infection, the regional authorities and the National Influenza Centre (NIC) were alerted at once, and oseltamivir therapy (75 mg twice daily) was initiated. On day 6 after admission, real-time RT-PCR was performed to detect avian IAV subtypes H5, H7 and H9, and a positive result was obtained for H9. On that day, nasal and throat swabs were collected from the patient, with only the throat swab testing positive for IAV. According to the national procedure [4,5], an aliquot of BAL sample was sent to the NIC where it was confirmed as an IAV subtype H9 (Table 2). The virus was isolated in Madin–Darby canine kidney (MDCK) cells (American Type Culture Collection (ATCC), CRL-2935) at both Fondazione IRCCS Policlinico San Matteo RRL and NIC.

On day 12 after admission, thoracocentesis was performed due to worsening pleural effusion, and the pleural fluid was tested for IAV, with negative result; the NPS collected tested positive for IAV H9. On day 16, a BAL was repeated and still tested positive for IAV H9, with a high viral load (Table 2). Over the following days, no notable clinical changes were observed. As of day 30, the patient was clinically stable and breathing on room air, although still febrile (Table 1).

## Public health actions

Epidemiological data were collected through direct interviews with the patient, their friends, household members and healthcare workers. In collaboration with the Italian Ministry of Health, potential close contacts on the return flight from Senegal were identified. During their time in West Africa, the patient stated that they lived in an urban household and that they were not in direct contact with animals, rural environments or individuals known to be unwell. They limited their activities to urban settings, including restaurants, supermarkets and local vendors, as well as making one visit to a coastal area. Contact tracing was carried out among the passengers of the return flight and the contacts in Italy according to the Italian Ministry of Health and the European Centre for Disease Prevention and Control (ECDC) guidelines [5,6]. This identified 13 individuals, including those seated next to the patient during the flight. These individuals were interviewed for early identification of their symptoms, possible modes of transmission and hygiene measures. A 14-day isolation at home from the time of exposure was recommended for those who had not worn personal protective equipment. Local health authorities also began monitoring these individuals for symptoms by daily phone calls. Eight of the 13 contacts were tested for influenza virus 7–10 days after the contact; all tested negative and received oseltamivir chemoprophylaxis. Six passengers seated adjacent to the patient and within two rows behind could not be traced.

## Molecular characterisation of influenza A(H9N2) virus

**Figure 1:**
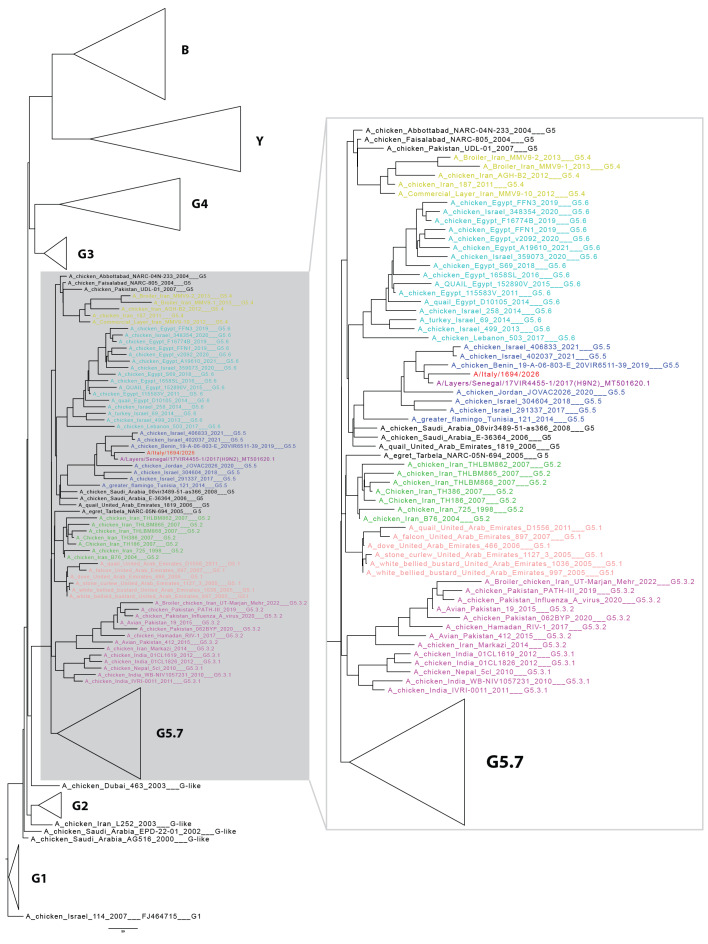
The maximum likelihood phylogenetic tree of influenza A viruses, 1978–2022 (n = 302)

**Figure 2:**
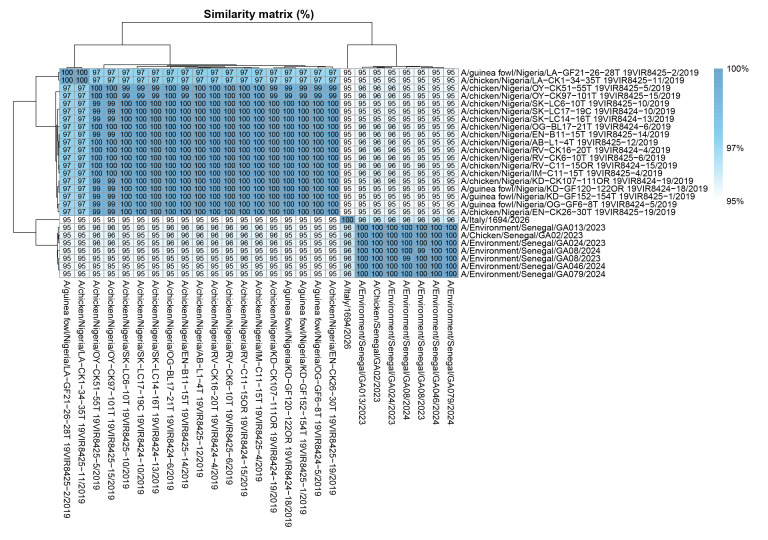
Nucleotide similarity matrix generated for the haemagglutinin segment against avian influenza A viruses, West Africa, 2019–2024 (n  = 26)

Whole genome sequencing was performed directly on the BAL sample using the Microbial Amplicon Prep—Influenza A/B kit (Illumina, San Diego, the United States (US)) and with a metagenomic shotgun short-reads sequencing. Resulting reads were mapped using MINIMAP2 [7] against the closest reference sequence, A/Oman/2747/2019. A nucleotide basic local alignment search tool (BLAST-N) analysis, on non-redundant databases, revealed the greatest genetic similarity with A/Layers/Senegal/17VIR44551/2017(H9N2). A maximum likelihood phylogenetic tree classified the virus as influenza A(H9N2) belonging to the G5.5 subclade and confirmed that the most closely related sequences were from Senegal (Figure 1). The sequence was uploaded onto GISAID (https://gisaid.org/) under the accession number EPI_ISL_20404890. The haemagglutinin (HA) sequence showed the highest nucleotide similarity (nearly 96%) with A(H9N2) strains identified in Senegal in December 2023. The similarity was also high (nearly 95%) compared with all H9N2 IAVs from West Africa isolated between 2019 and 2024 (Figure 2). Analysis of the amino acid sequences revealed that HA exhibited the Q226L substitution (H3 numbering), which is associated with enhanced binding to α2–6-linked sialic acid receptors. Additionally, it exhibited the HA-R156Q and HA-I212T mutations (H3 numbering), which have been linked to increased viral replication in mammalian and avian cells [8]. The HA cleavage site is typical of low pathogenic viruses. None of the main amino acid changes associated with adaptation to mammalian species were observed in PB2 (E627K, D701N) [9,10]. The NP-52N substitution, which is associated with evasion of a potent inhibitor of avian IAVs, was also identified [11]. Further amino acid substitutions observed in the internal protein genes of the A(H9N2) virus are presented in Table 3. No molecular markers associated with resistance to neuraminidase inhibitors were identified in the NA gene (including N2-H274Y) [12-14]. The PA-L28P substitution, which is associated with reduced susceptibility to baloxavir in human influenza A(H3N2) viruses, was identified [15].

The dataset included in the phylogenetic analysis was the subset of lineage sequences downloaded from the appendix 3 of Fusaro et al. (2024) [22]. Moreover, the most closely related sequence identified via BLAST (MT501620.1) was added. The tree was built using the best-fit model (GTR + F + I + R4), and the node support was assessed with 1,000 bootstrap replicates. The resulting phylogeny was refined and visualised using FigTree v1.4.4. The sequence identified in this paper is marked in red and the nearest sequence (MT501620.1) in violet. The nearest subclades are distinct, marked with different colours and named by the final letters of the sequence names. The tree was constructed using IQ-TREE v1.6.9 [23].

The sequences were deposited in GISAID (https://gisaid.org/).

**Table 3:** Additional mutations detected in internal protein genes of avian influenza virus A(H9N2) strain from a patient with recent travel history, Italy, March 2026

Protein	Mutation^a^	Phenotypic effects^a^
M1	T215A	Increased virulence in mice
	N30D	Increased virulence in mice
	I43M	Increased virulence in chickens, ducks and mice
NP	M105V	Increased virulence in chickens
	A184K	Enhanced interferon response
		Increased replication in avian cells
		Increased virulence in chickens
	E210D	Increased polymerase activity in mammalian cells
NS1	I106M	Increased viral replication in mammalian cells
		Increased virulence in mice
	C138F	Decreased interferon response
		Increased viral replication in mammalian cells
	V149A	Increased virulence and decreased interferon response in chickens
	P42S	Increased virulence and decreased antiviral response in mice
	D92E	Increased virulence in chickens, mice and swine
PA	S37A	Increased polymerase activity in mammalian cells
	N383D	Increased polymerase activity in avian and mammalian cells
	N409S	Increased polymerase activity and replication in mammalian cells
PB1	D3V	Increased polymerase activity and viral replication in avian and mammalian cells
	D622G	Increased polymerase activity and virulence in mice
PB2	V598T	Increased polymerase activity and replication in mammalian cells
		Increased virulence in mice
	L89V, G309D	Increased polymerase activity in mammalian cells
		Increased virulence in mice

^a^ Suttie A et al. 2019 [10].

The M2 gene contained the S31N mutation which is consistent with resistance to adamantanes [16]. No minority variants exceeding 15% were detected at any of the specified positions.

## Discussion

To our knowledge, this is the first reported human case of avian influenza A(H9N2) in Europe [2,17]. The detection of an unsubtypable IAV in the patient with severely weakened immune system prompted a thorough molecular investigation, including characterisation of the virus, which highlights the effectiveness of the diagnostic and surveillance system. The regional public health authorities identified, tested and interviewed 13 contact persons. Nevertheless, contact tracing is challenging when airline companies and tour operators are involved. Several individuals could not be traced; however, all those who were successfully traced and tested, returned negative results.

The genetic similarity of the virus to previously detected strains in West Africa suggests that the patient may have been exposed to the virus during their time in the region, despite reporting no direct contact with animals. The presence of molecular markers associated with human receptor binding further highlights the zoonotic potential of A(H9N2) viruses. However, there is currently no evidence of human-to-human transmission.

Notably, the initial NPS was negative for IAV, potentially due to inadequate specimen collection or a low viral load in the upper respiratory tract at the time of sampling. In this patient with weakened immune system, the infection was initially detected in the lower respiratory tract, as evidenced by BAL positivity. Later NPS positivity, however, was associated with high quantification cycle (Cq) values and suggested the detection of residual viral RNA rather than active replication in the nasopharynx [18].

## Conclusion

The potential for prolonged replication in patients with weakened immune systems raises concerns about the emergence of escape variants, emphasising the need for continued vigilance. This case underlines the importance of considering non-seasonal influenza viruses in patients with compatible symptoms and relevant travel history and highlights the added value of genomic characterisation in the public health response.

## Data Availability

Sequence generated in this study has been shared via GISAID, the global data science initiative, and can be retrieved under the accession number EPI_ISL_20404890.

## Members of the Collaborating Centres’ Study Group on Influenza

Department of biomedical sciences for health, University of Milan: Sandro Binda, Valeria Primache, Emanuela Matteucci, Arlinda Seiti, Michela Marcella Colleoni. Infectious Diseases Unit, Fondazione IRCCS San Gerardo dei Tintori: Elisa Colella, Alban Rugova, Selma El Ouardi, Giuseppe Lapadula, Viola Cogliandro, Guglielmo Marco Migliorino, Martina Comolatti, Eleonora Maria Beretta. Microbiology Unit, Fondazione IRCCS San Gerardo dei Tintori: Annalisa Cavallero, Sergio Malandrin, Iacopo Franconi, Dario Lafranceschina. National Influenza Centre, Department of Infectious Diseases, Istituto Superiore di Sanità: Marzia Facchini, Angela Di Martino, Sara Piacentini, Emanuela Giombini, Giuseppina Di Mario, Concetta Fabiani. Department of Prevention, General Directorate for Health, Lombardy Region: Simone Villa, Manuel Maffeo, Simona Scarioni, Marco Campana, Maria Virginia Coscarelli, Emanuele De Ponti, Anna Carole D’Amelio, Angela Ancona, Andrea Pedot, Federica Attanasi, Michela Viscardi. Regional Centre for Infectious Diseases, Lombardy Region (Ceremi) - Fondazione IRCCS Policlinico San Matteo: Gherard Batisti Biffignandi, Alessandro Ferrari, Guglielmo Ferrari, Stefano Gaiarsa, Antonio Piralla, Antonino Maria Guglielmo Pitrolo, Francesca Rovida, Raffaele Bruno, Valentina Zuccaro. Regional Centre for Infectious Diseases, Lombardy Region (Ceremi) - ASST Fatebenefratelli-Sacco: Andrea Gori, Riccardo Giorgi, Stefania Merli, Alberto Dolci, Alessandra Lombardi, Davide Mileto, Alessandro Mancon, Alberto Rizzo, Valeria Micheli. Health Protection Agency of Monza-Brianza: Chiara Ardemagni, Marianna Denova, Antonina Ilardo.
